# Model experiments on hydraulic properties around multiple piers with reproduced 3D geometries

**DOI:** 10.1038/s41598-022-24588-6

**Published:** 2022-11-19

**Authors:** Hirokazu Sato

**Affiliations:** Faculty of Life and Environmental Sciences, Shimane University, 1060 Nishikawatsu-Cho, Matsue, Shimane 690-8504 Japan

**Keywords:** Engineering, Civil engineering

## Abstract

The effects of multiple piers with 3D geometries on water levels during floods, as well as riverbed fluctuations and local scour after floods were examined via hydraulic model experiments. The Kintaikyo Bridge in Japan, a world-famous historical triple-wooden-arch bridge, was modeled at a scale of approximately 1/276. The number of model piers was set to four, as in the real bridge, and they were 3D printed and installed in the sand layer of an open channel. To provide adequate information on the pier installation conditions and hydraulic response, four cases were studied in the experiments, primarily considering present pier conditions; the previous conditions of the Kintaikyo Bridge were also considered. The experimental conditions in these four cases depended on whether foundations were present, the piers were skewed against the flow center, and the shape was spindle or similar to that of the present case. In the current bridge condition where foundations are present and there is no skew, water level rise, riverbed fluctuation, and local scour were suppressed. Furthermore, the statistical analysis of the results suggested that riverbed fluctuations are more stable after flooding. The two piers with foundations had smaller scour areas than those without. Further, those with foundations generally had lower mean scour at the pier perimeters. Regarding those without foundations, there were no differences in riverbed fluctuations or local scour due to the skewed pier angle. In addition, the representative length of the pier with a 3D geometry was determined by dividing the projected area of the pier below the initial sand surface in the main flow direction by the pier height, and the relationship between the Froude number or the mean water depth and the maximum scour depth was investigated.

## Introduction

Riverbed fluctuations, including localized scouring, have been extensively investigated for river disaster prevention, environmental conservation, landscape protection, and so on. In particular, scouring around piers is a complex hydraulic phenomenon. However, the measurement and simulation techniques used to investigate it have become more sophisticated over the past few decades^1^. Classic hydraulic model experiments have been conducted on single piers with simple structural geometries. Cylindrical^2,3^, typical rectangular^4,5^, and specific bridge-pier geometries^6^ have been examined. Jain et al.^7^ conducted experiments on the mixtures of riverbed materials at pier installation sites. Rasaei et al.^8^ experimentally confirmed the scour in piers installed in the river meandering. Many computational models have also been applied to single piers: using large eddy simulation, a turbulence model^9^; the method using energy balance theory^10^; and a statistical model that attempts to extend existing models while quantifying their bias and uncertainty^11^.

Because actual bridges have multiple piers, including abutments, these have also been studied. The effect of installing piles at the bottom of the piers on the riverbed has also been investigated. For example, 3D numerical simulations of two linearly arranged piers with simple cylindrical geometries^12,13^, as well as 3D simulations of three linearly arranged piers with a similar geometry^14^ have been conducted. Yilmaz et al.^15^ modeled two cylindrical piers in tandem and conducted experiments. Mehta and Yadav^16^ examined the hydraulic stability of the Sardar Bridge over the Tapi River in India, which has closely arranged multiple piers constructed in parallel. From their experimental findings, Oben-Nyarko and Ettema^17^ concluded that the effects of piers and abutments arranged in close proximity should also be considered. Further, Ghodsi et al.^18^ provided experimental results for scour in complex piers, including those with a column and pile cap.

In addition to local scour, the surrounding riverbed fluctuations are greatly affected by the degree of skewedness of the piers to the river flow, in addition to the shape, structure, and number of installed piers. In the medium to long term, the conditions of the riverbed over large areas of the river could change significantly. Yang et al.^19^ experimentally investigated the scour on a single elongated rectangular shaped slab pier skewed into a shallow stream. Through experiments on a thick rectangular pier, Kadono et al.^20^ showed that the scour area is enlarged when skew is toward the river flow, considering the collapse of a railroad bridge. Sato^6^ experimentally investigated the effect of a skewed spindle shape on the scour and water level changes. Fael et al.^21^ tested rectangular and several other pier geometries, including group piles, and discussed the equilibrium scour depth. The relationship between skewed river structures and scouring is presented in studies other than those on piers. For example, the scouring mechanism when a spur dike—a structure protruding from a riverbank into a river stream—is skewed has also been investigated^22,23^.

Scouring in the part of a river where water does not normally flow, for instance, piers on the flood plain in compound channels and the bank, has also been investigated^24,25^. Wu et al.^26^ investigated scouring at square and semicircular abutments in an ice-covered channel. Namaee et al.^27^ detailed the differences in scour between two adjacent circular piers, with and without ice, through experiments and using 3D numerical models. Further, studies have been conducted on riverbed fluctuations, as well as scour, in the absence of piers and other structures. Mohammad-Hosseinpour et al.^28^ determined the effects of meandering curvature and gravel pits interactions on maximum scour depth. Ma et al.^29^ conducted experiments on scour and deposition around notched groins to maintain navigation functions while taking into account the river ecosystem and analyzed the suitability of the habitat for fish.

Unique approaches to scour have emerged in recent years, such as applying machine learning to computational models^30–32^ and exploiting the vibrations in bridge ancillary structures for on-site scour prediction and monitoring^33–35^. There are also some studies on countermeasures for scour. Wang et al.^36^ and Valela et al.^37^ examined the effectiveness of antiscour collars and riprap placement for cylindrical piers, respectively.

**Figure 1:**
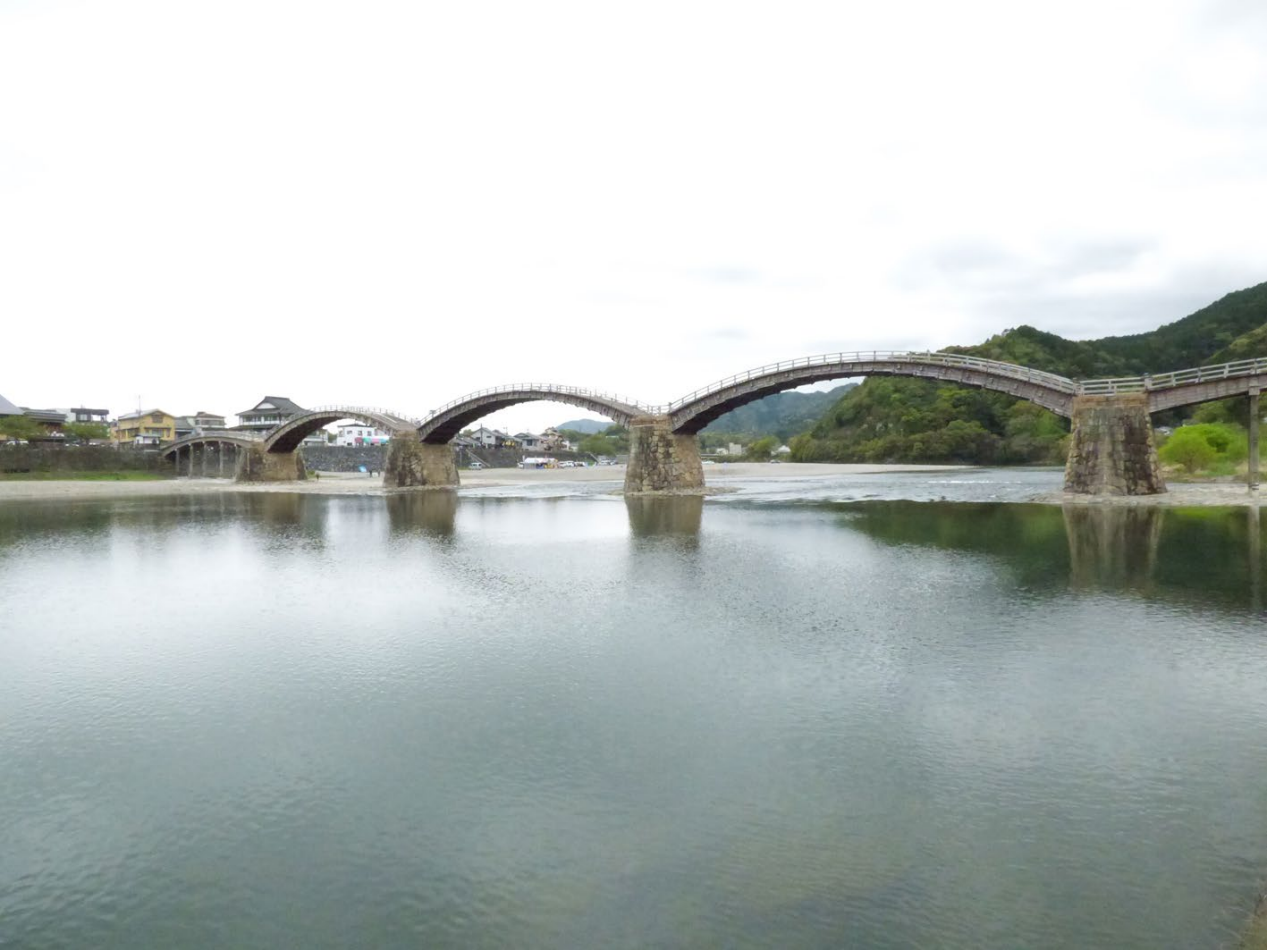
Kintaikyo Bridge.

In reality, the piers often undergo complex three-dimensional changes, and they are often equipped with ancillary structures such as foundations. In multispan bridges, multiple piers are installed. Moreover, they may be skewed against the river flow. Indeed, reviewed studies, whether experimental or simulation, have provided valuable academic insights into these individual conditions. For example, for a single pier, such as cylindrical^2,3,5,7–9^, rectangular^4,5,10^, or other special shapes^4–6^, the flow field, riverbed field, and bed material, among others are the main factor influencing scouring. Regarding multiple piers, however numerical results suggest that, in addition to these basic factors, the placement and number of piers may influence the scour characteristics by affecting the flow field^12–16^. Notably, structures, such as piles, placed below the bottom of the pier may influence the scour depth^17,18^. In addition, the degree of scour is more severe when the pier is skewed relative to the center of flow, regardless of the installation or hydraulic conditions^6,20,21^. In practice, a combination of several conditions affect the pier structure rather than the independent action of single factors. However, the relationship between piers installed under such varied conditions and hydraulic phenomena has not been sufficiently explored. In this study, hydraulic model experiments were conducted on the Kintaikyo Bridge (at the Nishikigawa River, Japan, Fig. 1), which has a complex combination of such conditions, considering its installation history. This study focused on the effects of differences in pier structure and installation conditions on water level fluctuations during floods, in addition to postflood riverbed fluctuations and local scour. The Kintaikyo Bridge is a five-span bridge: a central section has triple wooden arches, making it unique worldwide, in addition to its cultural heritage value^38,39^. Moreover, it has four piers, and their geometries are unique and complex (detailed in the in the section titled “Pier geometries”). Using this as a model case not only serves the aforementioned academic interests, but also contributes to elucidating the authenticity of its cultural heritage. Therefore, this study is distinct from previous studies as it aims to elucidate the value of bridges from engineering and cultural-heritage perspectives.

## Materials and methods

### Hydraulic model experiments

**Figure 2:**
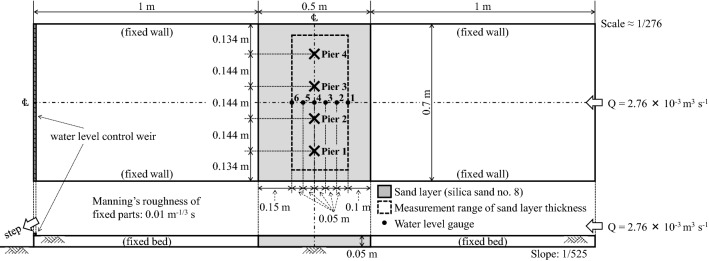
Experimental apparatus.

A straight open channel with a rectangular cross section was used in the hydraulic model experiment, with a sand layer in the center of the channel. Four piers were installed in this sand layer, and a steady flow rate was applied (Fig. 2). Moreover, clear water was used, and the width of the channel was 0.7 m. The geometric scale was 0.7/193.31 ≈ 1/276, based on the 193.31 m width of the Nishikigawa River where the Kintaikyo Bridge is installed. Froude’s law was used to determine the dynamic similarity of flow, and Shields law was applied to sediment motion. The length of the channel is assumed to be 1.5 m, and the sand layer is 0.5 m long and 0.05 m deep. Notably, this is the depth at which the 10 m foundation of the actual piers can be fully penetrated under the experimental scale above.

The target flood magnitude was assumed to be a peak discharge of 3500 m^3^ s^–1^, which corresponds to a medium to large flood at the Kintaikyo Bridge site. The discharge in the experiment was calculated to be 2.76 × 10^–3^ m^3^ s^–1^, as given by Froude’s law. The preliminary experiments indicated that the duration of one experiment was 90 s for all cases after the water reached the piers. This corresponds to approximately 25 min in real time, according to Froude’s law. The peak water depth at this time was assumed to be 5.3 m on the real scale; it could not be determined conclusively due to the influence of the downstream water level during flooding, including the tidal level and the riverbed conditions. In the experiment, the downstream weir height was adjusted to achieve a water depth of 0.019 m at the center of the pier installation section when there were no piers. The Froude number is approximately 0.47, so the flow is subcritical. Therefore, the choking phenomenon caused by floods passing through the piers does not occur. Even though the water-level conditions downstream of the piers, such as tidal fluctuations at the river mouths, may cause faster flow for the same flood discharge, a Froude number of approximately 1 and a flow close to critical are unlikely. Notably, no choking phenomenon is observed in the flood photographs of the Kintaikyo Bridge, presumably due in part to the Kintaikyo Bridge having sufficient clearance. The roughness coefficient of the fixed parts except for the sand layer is 0.01 m^–1/3^ s.

**Figure 3:**
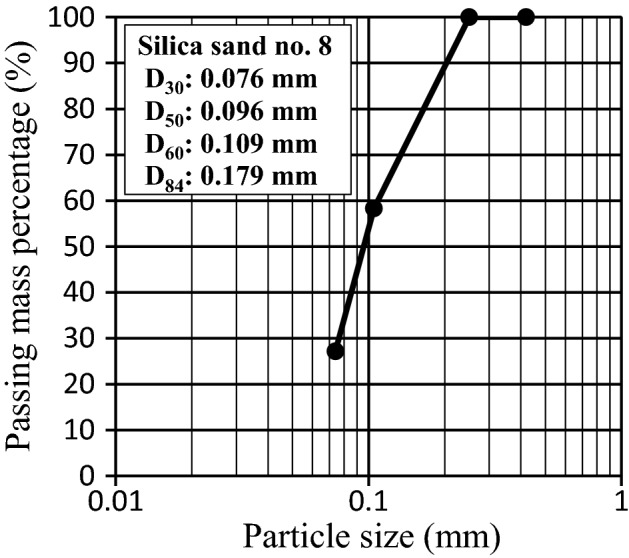
Particle size distribution used in experiment.

The riverbed slope near the Kintaikyo Bridge is relatively steep, 1/500–1/600, and the local riverbed material consists of sand and gravel ranging from a few mm to tens of mm. The channel slope was set to 1/525 in the experiment, and silica sand no. 8 with a median particle size of approximately 0.1 mm was used to maximally preserve the physical properties of the local riverbed material (Fig. 3). Assuming the specific gravity of sand in water to be 1.65, the Shields parameter relative to the median particle size was approximately 0.22 under the experimental conditions. The mean local sand and gravel size converted from this value is approximately 2.65 cm, which is consistent with the aforementioned range of the particle size distribution in the local riverbed. Neither the Froude number nor the Shields number exhibits a large deviation from the predicted hydraulic properties both in the field and experiments, which is reasonable considering that the riverbed fluctuates violently during medium-to-large floods. The boundary Reynolds number was approximately 1.273 in the experiment and 5841 in the field; notably, the mean water temperature was approximately 8 °C in the experiments. Although a gap exists between the two boundary Reynolds numbers, the corresponding the critical Shields parameters are approximately 0.1 and 0.06, respectively. Because the Shields number is 0.22, adequate sediment transport was assumed in both the experiment and field with regard to the given flood magnitude. However, careful experiment design is required when the flood magnitude is changed or the sediment type and particle size of the riverbed in the experiment and field do not match (especially the presence or absence of cohesion). Both the wet and the dry densities were approximately 1.35 g cm^–3^, and the sand used was in a naturally dry state with a moisture content ratio of approximately 0.18%. This was spread in the sand layer area without rolling compaction, and saturation before the flow water arrived was ensured. The experiment was repeated five times for each case.

The water level was measured during the experiment (six points, see Fig. 2), whereas the sand layer thickness and scour depth around piers were measured after the experiment (described later). Ultrasonic water level gauges were used at a sampling rate of 100 Hz to measure the water level. Depth and point gauges were used to measure the sand layer thickness and scour depth. Table 1 summarizes the corresponding parameters of the prototype and model.

**Table 1:** Experimental specifications.

Hydraulic conditions	Prototype	Model
Water surface width (m)	193.31	0.7
Geometric scale	0.7/193.31 ≈ 1/276	
Peak discharge (m^3^ s^−1^)	3500	2.76 × 10^–3^
Peak water depth (m)	5.3	0.019
Peak mean flow velocity (m s^−1^)	3.416	0.206
Manning’s roughness coefficient (m^−1/3^ s)	0.026	0.010
Bed slope	1/500–1/600	1/525
Duration of flood peak	25 min	90 s
Froude number	0.47	
Shields parameter	0.22	
Boundary Reynolds number*	5843	1.273
Reynolds number*	1.235 × 10^7^	2691

*Calculated using kinematic viscosity coefficient at water temperature of 8 °C.

### Pier geometries

**Figure 4:**
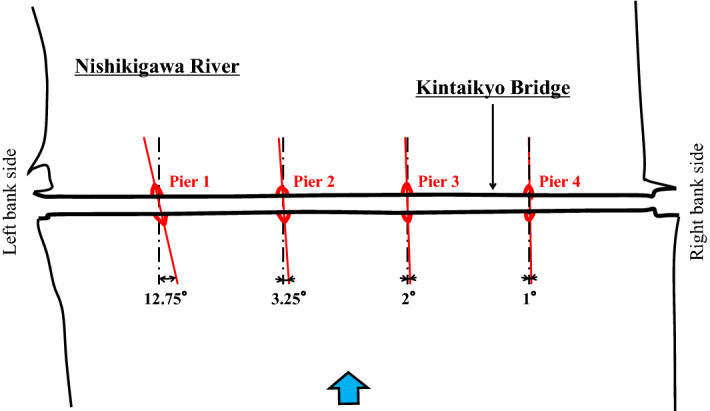
Installation angles of piers prior to loss by flooding in 1950: Redrawn from a figure in Ono^40^.

The Kintaikyo Bridge has 10 m foundations for all four piers and is not skewed against the flow of the Nishikigawa River. The bridge was rebuilt after being washed away by a flood in 1950, and its appearance has been maintained to the present day. Prior to that, the bridge had no foundations and was skewed against the flow of the Nishikigawa River^40^ (Fig. 4). It has long been theorized that the pre-1950 piers were completely spindle shaped (e.g., JACAM^41^). However, recent surveys and studies have objected to this view, and some scholars believe that the shape is closer to the present one^42^.

Considering the interesting changes in the installation conditions of the Kintaikyo Bridge, this study attempted to make comparisons under the following four experimental conditions. The first is the current situation where each pier has a foundation without skew, hereafter referred to as “current.” The second is the case where the skewed condition has not been corrected and is hereafter referred to as “current (skewed)”. The third is the old shape without a foundation and skewed, and it is assumed to be spindle shaped. Hereafter, it will be referred to as “old (spindle type).” The fourth is similar to the third, but it assumes a shape similar to the current one, hereafter referred to as “old (current type).” In the actual Kintaikyo Bridge, groundsills were installed around the piers to prevent scour, but these were not installed in the model. Instead, pier stability was measured by inspecting changes in the riverbed and the degree of scour, which provided general information.

**Figure 5:**
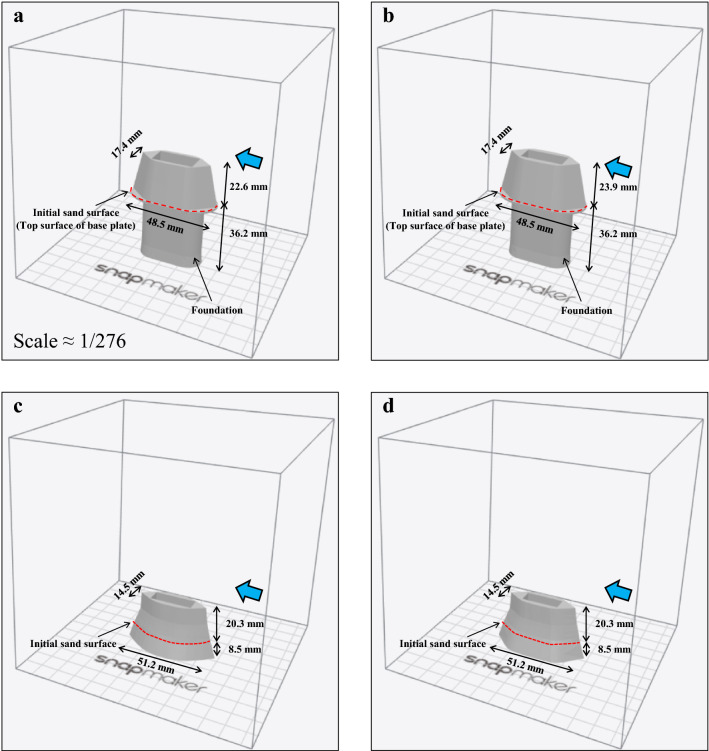
3D model of piers. (**a**) current piers 1 and 4, including skewed; (**b**) current piers 2 and 3, including skewed; (**c**) old (spindle type); (**d**) old (current type). Reproduced using CAD data provided by Iwakuni City and from JACAM figures ^41^.

**Figure 6:**
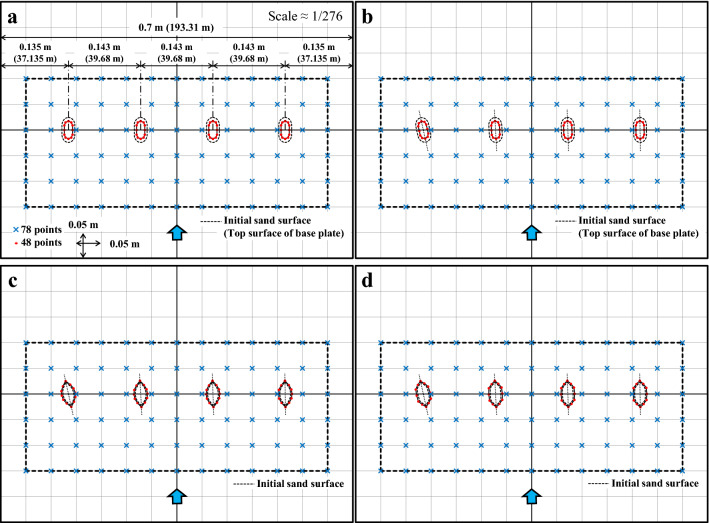
Measurement points for sand layer thickness and scouring around piers. (**a**) current; (**b**) current (skewed); (**c**) old (spindle type); (**d**) old (current type).

All of these configurations are complex, with or without foundations, and exhibit 3D variations in shape. Therefore, these were reproduced using a 3D printer (Fig. 5). The current, including skewed, were reproduced from CAD blueprints (provided by Iwakuni City), and the old (spindle type) was traced from JACAM figures ^41^. JACAM stands for The Japanese Association for Conservation of Architectural Monuments. The old (current type) had the same width and length as the old (spindle type), and that shape was assumed to be similar to that of the current. Below the sand surface in the two cases of old, the exposed pier above the initial sand surface had a stretched shape. The depth was taken as 2.35 m on average (8.5 mm in the experiment). As for the current, a thin base plate was sandwiched between the pier and the foundation, which was also reproduced in the model. Because the model was made of plastic, a hollow was provided in the center of the model and filled with iron sand to increase the weight. That the models remained stationary during the experiments was considered satisfactory, and their similarity to real piers in terms of weight was not taken into account. The installation intervals of the piers in the model were the same as those of the actual Kintaikyo Bridge, and 78 changes in sand layer thickness were measured around the piers. Additionally scour depth was measured at 48 points (12 points per pier) in the pier perimeters (Fig. 6).

## Results and discussion

### Water level change

Water levels were measured at a sampling rate of 100 Hz, and noise was processed using a 3 s median filter. The elevation datum for the water level was the channel bed (initial sand surface elevation) of water level gauge 4 (see Fig. 2) at the center of the pier.

**Figure 7:**
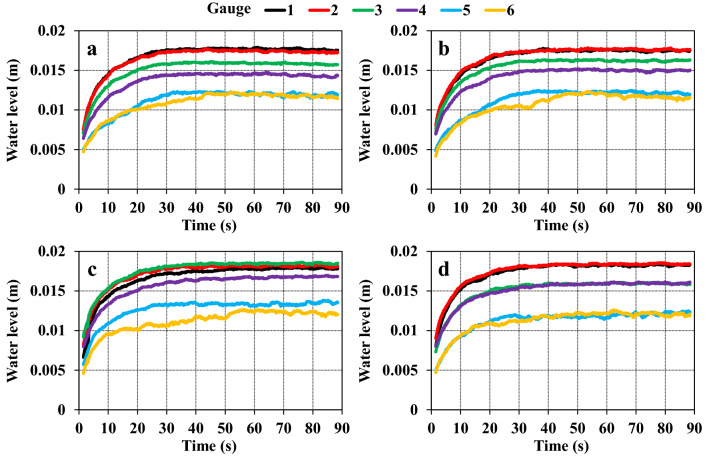
Temporal variations in water level. (**a**) current; (**b**) current (skewed); (**c**) old (spindle type); (**d**) old (current type). Water level datum: channel bed at gauge 4. Mean of five experiments.

Figure 7 shows the temporal variation in the mean water level from five repeat experiments for each of the four cases. Notably, in all experimental cases except for water level gauges 5 and 6 downstream of the bridge piers, a near steady state is reached at approximately the same time. This was 30 s after the water had been flowing for the current case. The steady state was reached after approximately 40 s for the other three cases. At gauge 6, which is the downstream-most point, the steady state is reached after a relatively long duration for all cases, whereas the slight fluctuations in the water level, such as noise, are slightly more intense. In each case, a sharp drop in water level was confirmed at the two downstream points unlike to the four upstream points, including the pier installation point. Except at the start and end of the experiment, the duration for the change in sand layer thickness could not be measured. Accordingly, the exact time that water depth changed was also not known, but these were presumed to be near the critical water depth (≈ 0.012 m) under the present experimental conditions. In other words, it was likely on the verge of becoming supercritical flow. This is due to relationship between the relative water level and that of the backwater upstream of the piers. In all cases, the water level is generally higher upstream throughout the duration of flow. However, for the old (spindle type), the water level is locally higher at gauge 3 immediately upstream of the piers, and for the old (current type), the water surface gradient at the two points near the piers is almost zero. It was not possible to determine if this phenomenon was caused by the lack of foundations.

**Figure 8:**
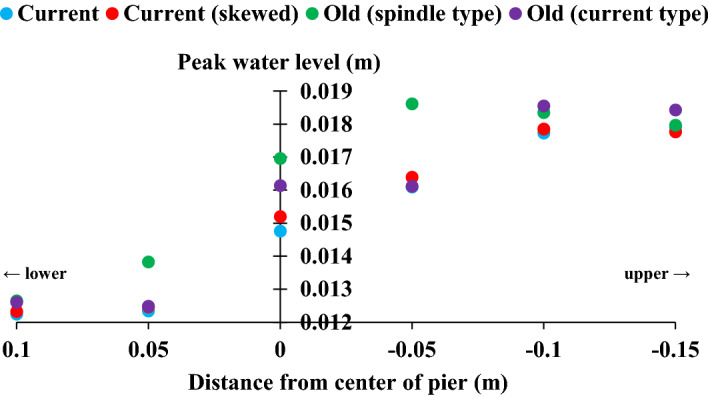
Peak water levels. (**a**) current; (**b**) current (skewed); (**c**) old (spindle type); (**d**) old (current type). Water level datum: channel bed at gauge 4. Mean of five experiments.

Figure 8 shows the mean peak water levels during the experiment for each case. Note that this does not necessarily correspond to the water level at the end of the experiment. Moreover, the temporal variation in the above water level results indicate that the peak water level is also larger overall in the old (spindle type). In particular, the peaks are clearly higher in the vicinity of the piers here than in the other cases. The three cases excluding that one generally show relatively close peak water levels regardless of the points of water level measurement, but the water level of the old (current type) is high at the center (gauge 4) of the pier installation location. Therefore, peak water levels around the piers were generally higher in the two cases that did not have foundations.

As shown in Fig. 5, the width of the structure reduces in the two cases with foundations as scour intensifies (width of piers > width of foundations), whereas it increases in the two cases without foundations as scour progresses. As a result, the water flow resistance increases as the sand surface decreases, and the peak water level may have also increased. A comparison between the two cases with foundations reveals that the current without skew has a lower peak water level. Presumably, the water flow resistance is lower in the absence of skew. It was not clear from these experiments how much the base plate between the pier and the foundation, which the two cases with foundation have, affected the change in water level.

### Fluctuation in riverbed and local scour

**Figure 9:**
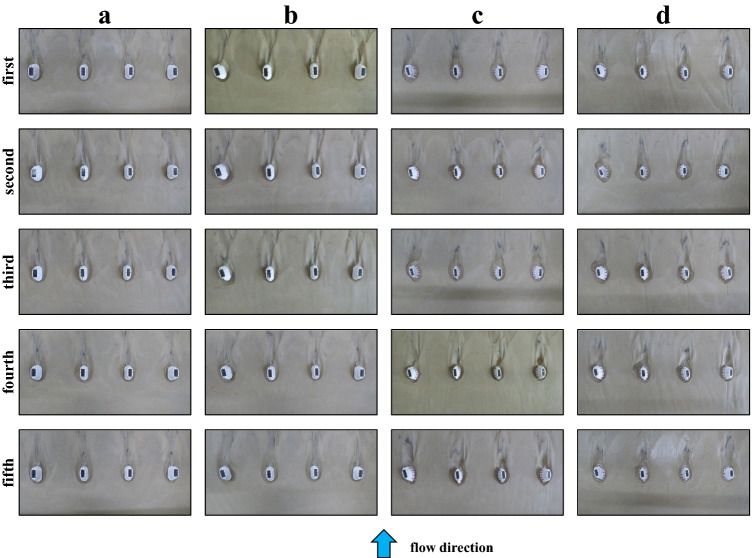
Photographs of sand layer and scour at end of experiment. (**a**) current; (**b**) current (skewed); (**c**) old (spindle type); (**d**) old (current type).

The condition of the sand layer around the piers at the end of the experiment is shown in Fig. 9, presenting the five experiments for each case. In the current, where all four piers are not skewed with respect to the flow, the sand layer changes, and scours are relatively even in all five experiments. The other three cases with skew revealed local scour at the periphery of the piers, especially in front (upstream) of the piers. Local scour is greatest in pier 1 (left side of the photos), where the skewed angle is greatest.

**Figure 10:**
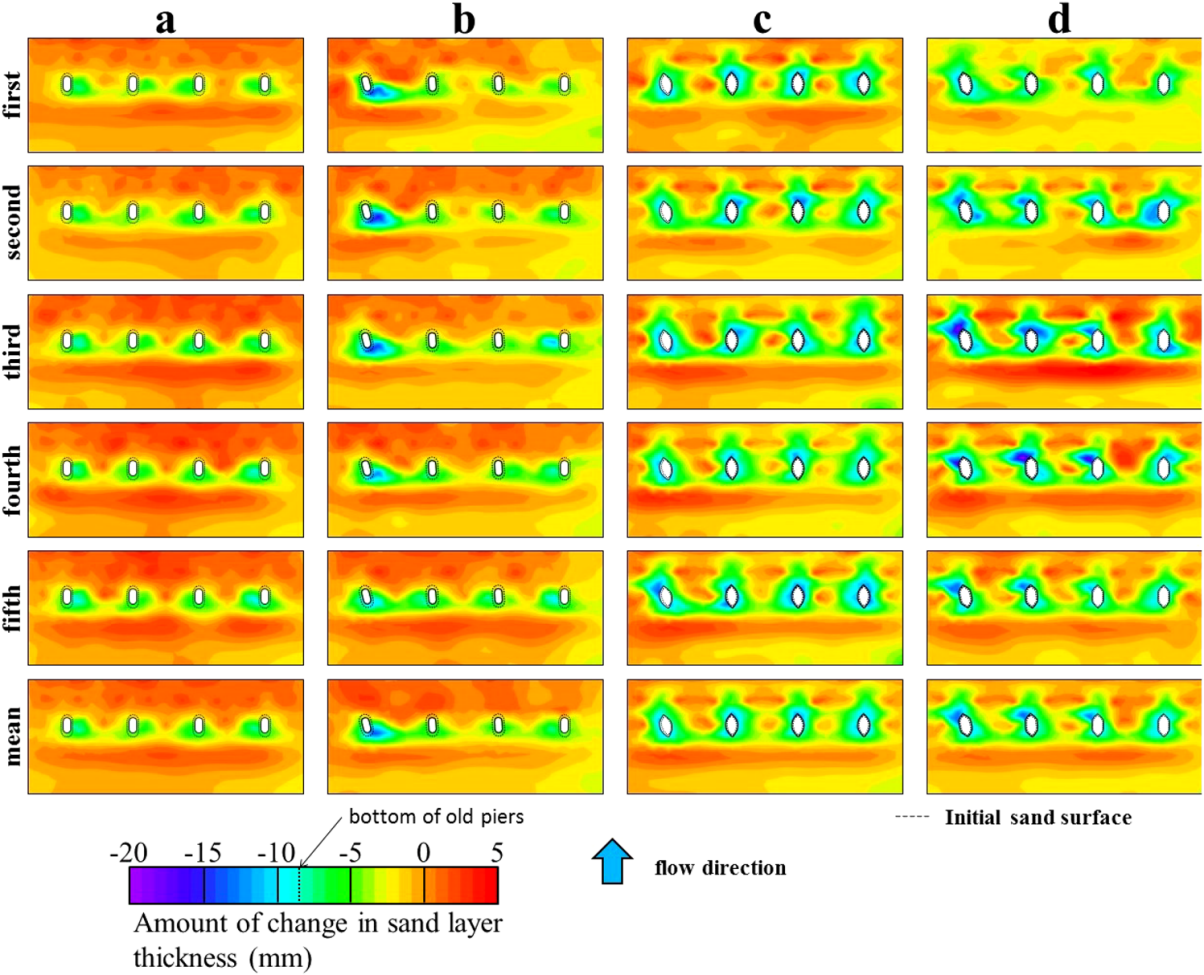
Changes in sand layer thickness after scouring around piers at end of experiment. (**a**) current; (**b**) current (skewed); (**c**) old (spindle type); (**d**) old (current type).

Figure 10 shows the results of the changes in this sand layer thickness and local scours, which were measured at the points shown in Fig. 6. The mean of the five experiments is also included in Fig. 10. The area is the measurement range for sand layer thickness. Quantitative validation shows that although there is a slight bias toward local scour in piers 3 and 4, the current without skew is generally uniform with changes in sand layer thickness and scour, both in the individual experiments and in the mean. The entire pier perimeter is scoured, but sedimentation can be seen at a small distance behind it. On the other hand, in the current (skewed) case, scouring is more severe in pier 1, where the skewed angle is the largest. This was confirmed in all five experiments. The second most severe local scour is seen in pier 2, where the skewed angle is the second highest, followed by less scour in piers 3 and 4, where the skewed angle is closer to 0°, and there is little difference. Moreover, in all four piers, scour is present at the periphery of the piers, but a sedimentary zone is formed further downstream.

In the two cases of old without foundations, both show similar sand layer thickness changes and local scours around the four piers, regardless of the degree of the skewed angle. However, the scours around and at the perimeter of the piers in the two cases with foundations were concentrated in front of the piers (upstream side), whereas in these two cases it extended to the rear of the piers (downstream side). Although the piers were not washed away under the experimental conditions, scour extended to the bottom of the piers in the no-foundation cases (−8.5 mm in the model, see Fig. 5), and maybe even below the bottom of the piers. This suggests that a portion of the water flow may reach the bottom of the piers. The flow passing through the bottom of piers may have accelerated scour, including the back of the piers, by encouraging the downstream movement of sand particles, especially the fine components, inside the sand layer. In some cases with foundations, the scour depth was even greater than −8.5 mm, but the foundation depth was 36.2 mm in the model scale. Therefore, local scouring was considered to be limited. In addition, as mentioned in the section titled “Water level change,” the two old cases have a three-dimensional pier geometry that increases in width as scour intensifies at the pier perimeters. This complicates the vortex structures around the piers as scour progresses and increases the resistance to flow. In the cases with a foundation, in contrast, the shape below the sand surface is almost constant, constituting another major difference from the cases without foundation. Another difference from the cases with foundations is the absence of a sedimentation zone behind the piers (from a comparison of (a) or (b) with (c) or (d) in Fig. 10), resulting in slight erosion. This difference in the formation of sedimentation zones is largely due to the difference in the water level gradient from the rear of the piers to the downstream side, that is, the difference in the flow velocity. Comparing the water levels of water-level gauge 4 at the center of the pier and gauges 5 and 6 downstream of it, shown in Fig. 7, suggests that the difference is larger and the flow velocity faster in the two cases without a foundation than in the two cases with a foundation during the entire duration of the water passage. However, because Fig. 8 plots the peak water levels, the water level recording times in each gauge are not exactly the same. Nevertheless, this figure analogizes the above-mentioned relationship between large and small water surface gradients. In addition, the cases with and without skewed piers have different skew angles, which becomes smaller from piers 1–4, whereas upstream of the piers, more erosion occurred on the side of pier 4. This may be attributed to the fact that, considering the cross-sectional distribution of water depth upstream of the pier, the water depth is greater on the side of piers 1, where the skewed angle is greater, whereas the flow velocity is lower than that on the side of pier 4.

**Figure 11:**
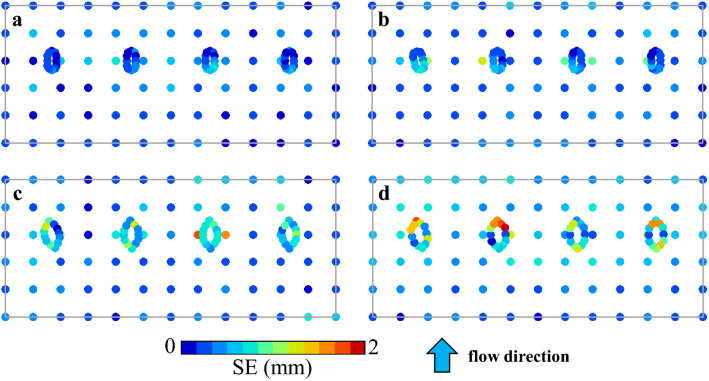
Spatial distributions of SE (standard error) for the five experiments at measurement points for sand layer thickness and scouring.

Each case was repeated five times under the same conditions, but the hydraulic model experiments on movable beds exhibited the dispersion tendency in the results. This is attributed to the sensitivity of the phenomenon to minute fluctuations in the flowing water supplied by the pump control and to minute nonuniformities in the nature and spreading of the sand used in the experiments. Mechanical and human errors may have also been present in measurement. Because understanding this dispersion provides reference information for similar hydraulic model experiments, the standard error (SE) for the five experiments measuring the variation in sand layer thickness, including local scour, is shown in Fig. 11. This means that the SE here represents the dispersion relative to the mean of the measured sand-layer thickness changes for the five experiments at the corresponding measurement points, including the periphery of the piers shown in Fig. 6.

Generally, both changes in sand layer thickness and local scour exhibited little dispersion between the two cases that have foundations. Specifically, there is no significant difference in the dispersion of changes in sand layer thickness around the piers; near the piers of the current (skewed) case, it is marginally larger, and the difference is not extreme. In the two cases without foundations, the dispersion in scour at the pier perimeters is large; however, the dispersion is not particularly noticeable in pier 1 in each case, which has a large skewed angle in both cases. The SE is large in piers 2 and 3 of the old (spindle type), and it is large in piers 2 and 4 in the old (current type).

Although the three-dimensional distribution of flow velocity could not be measured in the experiments, as mentioned earlier, the flow near the piers is more complex without foundations, and the scouring points change slightly due to subtle differences in flows and vortices in each experiment, which may cause the dispersion. Presumably, such instability is alleviated by the presence of foundations. Therefore, the destabilization caused by skewedness, which is the difference between the two cases with foundations, is also offset by the foundations. Accordingly, even in the same river, slight differences in flood and river conditions would cause significant differences in the riverbed fluctuations and local scour, depending on whether the bridge piers have foundations. Therefore, as long as the Kintaikyo Bridge remains in its present structural configuration, predicting changes in the riverbed is relatively easy, and countermeasures can be taken each time a flood occurs.

The maximum scour/erosion depths and sedimentation heights for each case, considering the dispersion of the experimental results, are shown in Table 2. It also lists the maximum values for each experiment, and the maximum mean values, SE and 95% confidential interval (CI) for the five experiments. Regarding the depths of scour/erosion, it is most suppressed in the current in each experiment and in the mean values. The mean value of old (spindle type) is the second smallest, but it differs from the current by 2.84 mm, which is approximately 78 cm in the actual scale. On the other hand, the current has the largest maximum sedimentation height; followed by the current (skewed); and then the two cases of old, which have similar values. Although scouring is a problem from the viewpoint of pier stability, the large amount of sedimentation is advantageous in locally availing sediment materials for restoring piers after flooding. For these cases with maximum values, the dispersion is also smaller for the two cases with foundations, regardless of scour/erosion or sedimentation, as is the overall trend.

**Table 2:** Maximum changes in sand layer thickness including pier perimeters.

Pier geometry	Maximum scour (mm)*		Maximum sedimentation (mm)*	
	Each expt.	Mean (SE) [95% CI]	Each expt.	Mean (SE) [95% CI]
Current	− 7.62	− 9.44 (0.627) [− 11.180, − 7.699]	3.25	3.35 (0.326) [2.450, 4.260]
	− 8.52		2.80	
	− 10.32		3.56	
	− 10.24		4.01	
	− 10.75		3.96	
Current (skewed)	− 14.17	− 14.36 (0.617) [− 16.073, − 12.647]	2.59	2.88 (0.268) [2.140, 3.630]
	− 15.64		2.99	
	− 15.25		2.56	
	− 14.65		3.80	
	− 12.11		3.24	
Old (spindle type)	− 12.82	− 12.28 (0.742) [− 14.343, − 10.225]	2.98	1.19 (0.454) [− 0.072, 2.446]
	− 13.84		1.89	
	− 13.67		1.62	
	− 12.84		2.05	
	− 13.90		2.48	
Old (current type)	− 11.93	− 14.31 (1.635) [− 18.849, − 9.767]	1.39	1.20 (0.769) [− 0.934, 3.336]
	− 15.56		1.09	
	− 17.60		3.72	
	− 18.27		3.15	
	− 15.46		2.13	

*Mean, SE, and 95% CI at measurement points as well as the largest values in the five experiments are listed.

### Discussion

Thus far, we have reviewed the changes in water level and sand layer thickness, and the measurement results of local scour. In this section, we will focus on local scour at the periphery of the piers. Local scour is often discussed in relation to the pier geometry, and the corresponding flow characteristics. While pier width is used as a representative length for pier geometry, the geometry of bridges such as the Kintaikyo Bridge is complex and three dimensional irrespective of the pier arrangement. The presence of foundations further complicates this, and when skew to the flow center is included, simply using the pier width as a representative length results in a loss in generality. On the other hand, the upstream depth and mean velocity are generally used, which are not affected by the piers. Therefore, the representative length here was defined as the projected area of the area below the initial sand surface, including the skewed area, in the direction of the main flow divided by the height (depth) to the bottom of the sand surface. For the same shape, if skew is toward the flow center, the projected area increases, as does the representative length. Regarding the current and current (skewed), the area of interest is from the top of the base plate to the bottom of the foundation. According to this notion, the representative length is given by

1 $$D = A_{s} /h_{s}$$

where $D$ is the representative length of the pier shape, ${A}_{s}$ is the projected area of the structural portion below the initial sand surface relative to the mainstream direction, and ${h}_{s}$ is the height of the structural portion below the initial sand surface. Taking the maximum scour depth as ${Z}_{s}$, the dimensionless maximum scour depth becomes ${Z}_{s}/D$.

The target flow index is measured at gauge 1, which is the point farthest upstream from the pier installation. The water depth at the end of the experiment, obtained by subtracting the sand layer height from the measured water level at the end of the experiment, is ${h}_{0}$. The sand layer height at gauge 1 is the mean of 13 measured values in the corresponding transverse direction. The cross-sectional water surface gradient at gauge 1 was assumed to be 0.

**Figure 12:**
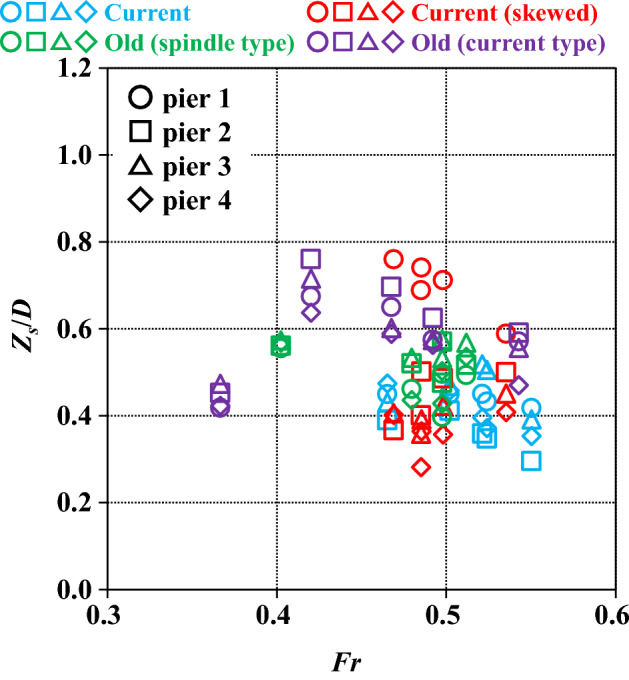
Relationship between maximum scour depth at pier perimeters and Froude number for the five experiments.

The Froude number ($Fr$ hereafter) a dimensionless measure of flow velocity, can be used to elucidate the relationship between $Fr$ and ${Z}_{s}/D$ (Fig. 12). In the figure, the pier numbers are distinguished by shapes such as circles and squares, and the experimental cases are divided by colors. The plots show the results of five experiments for each case. Overall, $Fr$ is large for the current and the current (skewed), and the dispersion is small. ${Z}_{s}/D$ is small for the current, large for the old (current type), and widely distributed in the current (skewed). In the two old cases, $Fr$ is scattered, but ${Z}_{s}/D$ is within a reasonable range. The ${Z}_{s}/D$ for each pier, current without skew, and the two cases of the old, suggest that the effect of different skewed angles for each pier is offset by the lack of foundations, as mentioned in the section titled “Fluctuation in riverbed and local scour”. This is consistent with the large ${Z}_{s}/D$ at pier 1 of the current (skewed) case.

**Figure 13:**
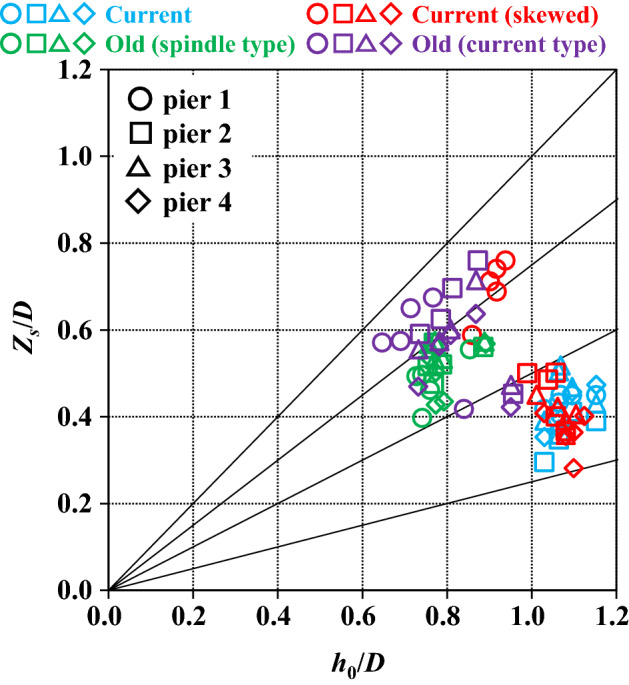
Relationship between maximum scour depth at pier perimeters and mean water depth for the five experiments.

The correspondence between ${h}_{0}/D$ and ${Z}_{s}/D$ is then considered, where ${h}_{0}$ is made dimensionless by applying $D$ (Fig. 13). The graph is organized in the same manner as above, but typical slopes (1, 0.75, 0.5 and 0.25) are inserted as visual aids. ${h}_{0}/D$ is large in the two cases with foundations because the width of the foundation is small. Therefore, except for a slight jump in ${Z}_{s}/D$ in pier 1 of the current (skewed), the overall results adequately reflect the differences in geometry, including the effects of foundations and skew. Although $Fr$ is commonly used to organize the results, the velocity distribution at the pier perimeters is more important than the mean velocity in explaining local scours. The water depth serves as a more universal index, and as far as the Froude law is applicable, the $D$ proposed here and the observed water level (water depth) can be used to adequately estimate the maximum scour depth for each pier. This is true even for a real river with multiple piers, complex structures, and skew.

The approach flow conditions (velocity and depth) at water-level gauge 1 considered here are almost identical because the same flow from the upstream side of the piers is considered in this study; however, the pier structures and installation conditions differ. In contrast, the magnitude of the scour varies with the pier conditions. Therefore, the non-dimensional index based on the representative length that reflects the pier geometry proposed here is important, and it allows for a universal arrangement of the relationship between approach flow and scour, including cases where the conditions are identical. In this study, the choking phenomenon did not occur under subcritical flow conditions, but the notion of the choking phenomenon affecting the scour of piers in rivers with critical or supercritical flows is plausible. In this case, because the spacing between piers is important, the projected area of the piers and height of the structural parts necessary for calculating the representative length could be raised above the sand layer. However, this will need validation in future experiments.

**Figure 14:**
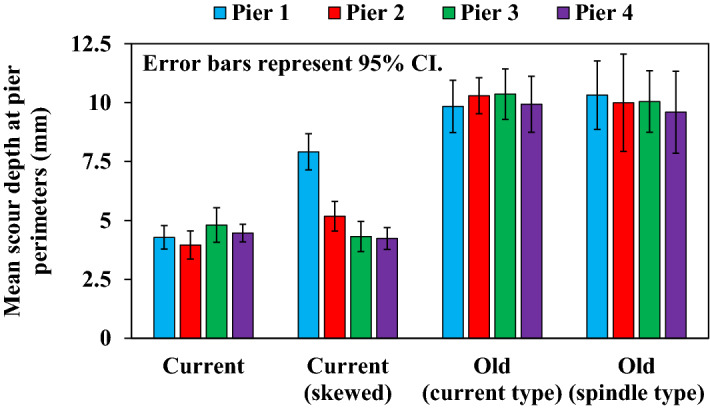
Mean scour depth at pier perimeters for the five experiments.

Although a hydraulic summary of the maximum scour depth is presented, we should also look at the overall scour conditions at the pier perimeters. The scouring depth was measured at 12 points per pier, and the mean of these was taken as the scouring depth of a given pier. Figure 14 shows the mean of the five experiments for the scour depth in each case. Error bars in the figure represent the 95% CI. This validates the difference in overall scour depth with and without foundations. In the current (skewed) case with foundations, the scour depth of the entire pier perimeter also responds to the degree of the skewed angle. The scours of the entire pier perimeter of the two cases without foundations are seemingly unaffected by the degree of the skewed angles, and it confirmed that the lack of foundations may have eliminated the effect of the skewed angles. The range of 95% CI revealed that the two cases without foundations have greater experiment-to-experiment dispersion here as well.

This study should be compared with others that investigate complex pier geometry, and its applicability to other sites should be investigated. Oben-Nyarko and Ettema^17^, and Ghodsi et al.^18^ studied scour; the piles with a pile cap were situated at the pier bottom, and the former even considered the interactions of abutments and piers^17^. Fael et al.^21^ tested a pile group (but not at the pier bottom) with and without skew. This study dealt with foundations, base plates, and skews, but these are similar in terms of the plurality of pier structures. The results corroborate our findings, suggesting that the scour processes and results differ from those for piers with simple construction and installation conditions. Although this study considered the foundation geometry of the Kintaikyo Bridge, the findings can be extended to different foundation geometries, such as the current (skewed) case, where the projected shape of the piers is skewed with respect to the flow. Therefore, the method proposed in this study for calculating the representative length of piers below the sand surface can be universally applied to different extents for different types of foundations.

## Conclusions

Hydraulic model experiments were conducted using four piers modeled after the Kintaikyo Bridge, which has three-dimensional geometries. The water level and riverbed fluctuations, as well as local scours around the piers, tended to be lower when the piers had foundations. When the piers were skewed against the flood flow, the water level rise and scour were greater. Specifically, scouring was localized for sufficiently deep foundations, whereas scouring presumably progress to the bottom of the piers when the piers were buried only to a shallow sand layer without foundations. Water channels formed on a part of the pier bottom, and eventually the entire pier was scoured. However, even with foundations, scour increased with increasing skew to the flow center. The piers with foundations exhibited less statistical dispersion in the experimental results for all evaluations of scour/erosion and sedimentation.

A method of determining the geometrical representative length of piers, which is important when considering the relationship between the maximum scour depth and flow velocity or water depth, was established to accommodate complex 3D changes in the shape of the piers, including cases where piers are skewed with respect to the flow center. Therefore, the relationship between $Fr$ and ${Z}_{s}/D$ and ${h}_{0}/D$ and ${Z}_{s}/D$ was elucidated. ${h}_{0}/D$ and ${Z}_{s}/D$ indicators were more versatile and reasonably explained the relationships between complicated factors, such as presence or absence of foundations or skew, and maximum local scour. In other words, foundations reduce scour, which increases with larger skewed angles. On the other hand, the absence of foundations may increase scour sufficiently to offset the effect of the presence or absence of skew.

The results of this study suggest that in rivers with sand or gravel beds, where the Froude number is in the critical flow range—implying that the choking phenomenon at the piers is unlikely to occur—a foundation with sufficient depth can reduce scour and unstable riverbed fluctuations around piers. In particular, the method considering the representative length, as proposed in this study, is effective for evaluating scour at bridge sites that fit these conditions. Thus, it can replace existing methods that simply use pier width and height as indicators.

As a limitation during the experiments, an environment where the flow velocity can be measured on a microscale was not prepared. In future work, a precise 3D turbulence model will be built and the data obtained used for model calibration, acquiring detailed information on flow velocity, and extending the model to simulations at actual bridge piers in different rivers. This project will contribute to academic research interests in sediment hydraulics and will rationalize the presence of piers in the Kintaikyo Bridge, which is considered a valuable cultural heritage site.

## Data Availability

Please contact the corresponding author for data requests.
